# Mobilizing to overcome tobacco industry interference in lawmaking

**DOI:** 10.18332/tid/135637

**Published:** 2021-06-07

**Authors:** Traci Kennedy, Nichelle Gray, Gabrielle Ballweg

**Affiliations:** 1 Americans for Nonsmokers’ Rights, Berkeley, United States; 2 American Nonsmokers’ Rights Foundation, Berkeley, United States; 3 Action on Smoking and Health, Washington, United States

**Keywords:** tobacco industry, advocacy, policy interference, intersectoral partnerships, coalition activism

Tobacco industry interference continues to hinder the implementation of stronger tobacco control policies. Fortunately, tobacco control advocates have remained vigilant, countering these measures, and mobilizing best and innovative practices to strengthen public health policies. Americans for Nonsmokers’ Rights (ANR) and the ANR Foundation have taken on Big Tobacco and their strategies to undermine smoke-free air and other public health protection since their inception over 40 years ago. Similarly, Action on Smoking and Health (ASH) is America’s oldest anti-tobacco organization, dedicated to a world with ZERO tobacco deaths. Because tobacco is the leading cause of preventable deaths worldwide, ASH supports bold solutions proportionate to the magnitude of the problem. Including changing social norms to end the age of the cigarette and demanding respect for basic human rights and protection against the tobacco industry and their products.

Operating in the shadows has been Big Tobacco’s *modus operandi*, but their conviction as racketeers in the federal RICO case exposed the tobacco industry’s primary objective: profit over people^1^. For decades, their scientists and researchers undermined the science of secondhand smoke and created increasingly addictive products^2^. Today, rather than relying on front groups for political coverage, the industry is directly drafting legislation to create carve-outs and keep smoking indoors.

Tobacco industry interference deepens existing gaps in smoke-free protections particularly for the LGBTQ2+ population, people of color, and those that work in the hospitality industry^3^. Companies like Altria and Phillip Morris tout claims of addressing equity but continuously fight equitable measures to protect these same people^4^.

In state houses across the country, broader public health measures related to mask-wearing and the COVID-19 response are also under attack by long-time tobacco industry allies like the American Legislative Exchange Council^5^. This action is a standard mechanism to keep smoking indoors, which is impossible while properly wearing a mask.

Direct tobacco industry interference has also been apparent in California’s SB38 and SB793 bills. In 2019, a California state senator introduced SB38, which would have imposed a flavor ban on tobacco products. The bill initially passed through the California Senate Health Committee with momentum; however, the Hookah Chamber of Commerce derailed the bill. In response, the chair of the California Senate Appropriations Committee inserted amendments including exemptions for hookah products, among others. These amendments greatly impacted the ability to pass a comprehensive policy. Additionally, this action prompted other legislators in cities like Burbank, California, to pass ordinances with exemptions for hookah^6^. Hookah exemptions are a clear example of how the tobacco industry can pivot their focus to weaken legislation^7^.

In 2020, the California bill was reintroduced as SB793 and again sailed through the State Senate Health Committee. However, the Senate Appropriations Committee chair opposed SB793, so to move forward, hookah had to be exempted once again^8^. In the State Assembly, due to COVID-19, the bill was only heard by the Health Committee^8^. To sway assembly members, the tobacco industry increased spending to promote claims that police interactions with people of color and racial profiling would increase with menthol prohibition^9^. After additional amendments to exempt premium cigars and piped tobacco products, SB793 managed to make it out of the Committee^8^. Tobacco companies then leveraged these amendments to construct attack advertisements, claiming that SB793 was deliberately targeting African American and Latino communities by making cigar exemptions for the rich^10-12^. Big Tobacco spent over $1 million in August 2020 on television and radio advertisements attacking SB793 and funded grassroots and Neighborhood FORWARD rallies, calling SB793 racist^11,13^.

After going through the Appropriations Committee, SB793 passed the Assembly 58 to 1, and the California governor signed it into law^8^. Predictably, the tobacco industry began gathering signatures immediately for a referendum. In California, a law can be overturned by asking voters to affirm the legislation on the next statewide general election ballot^14^. Within a few weeks, tobacco companies had spent $21.1 million to gather enough signatures to place the measure on the ballot: forcing the re-consideration of SB793 in front of voters in 2022^15^ and demonstrating the lengths to which the tobacco industry will go and their increasingly sophisticated tactics.

Throughout these industry attacks in California, tobacco control advocates made a sustained effort to increase genuine grassroots support, culminating in the sign-on of 150 public interest groups, labor organizations, and tobacco control and public health NGOs^13^. Advocates engaged African American leaders across the state for advertisements, religious leaders showed up at hearings, and a congresswoman wrote an op-ed countering industry arguments^16^. The tobacco control community also responded with advertisements thanking members of the state legislature for being heroes to healthy kids.

It is clear that tobacco control advocates must recognize the massive impact of non-traditional partners, especially at the local level. By fostering intersectoral and community partnerships, the tobacco control community can counter these new, more aggressive, industry methods. For example, in 2018, the Beverly Hills City Council proposed an ordinance to remove tobacco products from all store shelves^17^. It was successful in part because of local community involvement. Lobbyists from the tobacco industry were present during the early ordinance meetings, but because of strong groups like parent teacher associations (PTA) and other non-traditional tobacco control partners, the City Council recognized that they needed to act on this issue. On 1 January 2021, Beverly Hills became the first jurisdiction in the US to eliminate the sale of commercial tobacco^17^.

When smoke-free school initiatives gained momentum in Rhode Island, the tobacco industry began pushing new products, including e-cigarettes, and tobacco control advocates realized the necessity to update smoke-free policies^18^. Several Rhode Island school districts were approached by the tobacco industry – JUUL in particular – to include industryled educational programs into their curricula^19^. Through coordination among local corporate sponsors, students, parents, first responders, and healthcare providers, advocates and stakeholders created toolkits to educate schools and the public to identify these industry-led programs^20^. They were able to quickly respond by collaborating to design signage and toolkits throughout the state. This effort was successful because of the mobilization of youth and adult advocates.

These forms of authentic engagement and coalition activism have been fundamental to collective tobacco control successes, especially collaborative efforts where adult and youth voices hold the same weight throughout the process.

It is essential for tobacco prevention advocates and public health professionals to engage in discussions on tobacco industry interference. The tobacco industry interference examples above prove that Big Tobacco is not letting the COVID-19 pandemic slow them down or stop them. Advocates must mobilize action to make public health the loudest voice in the local, state, and federal government, combating interference and promoting common-sense public health policy.
